# Overuse of medical care in paediatrics: A survey from five countries in the European Academy of Pediatrics

**DOI:** 10.3389/fped.2022.945540

**Published:** 2022-09-13

**Authors:** Lina Jankauskaite, Yevgenii Grechukha, Kristin Avranden Kjær, Marina Mamenko, Britt Nakstad, Ivanna Romankevych, Sara Schnyder, Joel Selvakumar, Sandra Trapani, Sandra Daniliaviciene, Arunas Valiulis, Corinne Wyder, Ketil Størdal

**Affiliations:** ^1^Department of Paediatrics, Lithuanian University of Health Sciences, Kaunas, Lithuania; ^2^Institute of Physiology and Pharmacology, Lithuanian University of Health Sciences, Kaunas, Lithuania; ^3^European Academy of Paediatrics, Brussels, Belgium; ^4^Department of Paediatric Infectious Diseases and Paediatric Immunology, Shupyk National Healthcare University of Ukraine, Kyiv, Ukraine; ^5^Faculty of Medicine, University of Oslo, Oslo, Norway; ^6^Shupyk National Healthcare University of Ukraine, Kyiv, Ukraine; ^7^Ukrainian Academy of Paediatric Specialties, Kyiv, Ukraine; ^8^Division of Paediatric and Adolescent Medicine, Oslo University Hospital, Oslo, Norway; ^9^Department of Paediatrics and Adolescent Health, University of Botswana, Gaborone, Botswana; ^10^Miller School of Medicine, Jackson Memorial Hospital, University of Miami, Coral Gables, FL, United States; ^11^Division of Paediatric Emergency Medicine, Department of Paediatrics, Inselspital, Bern University Hospital, University of Bern, Bern, Switzerland; ^12^Department of Paediatric and Adolescent Health, Akershus University Hospital, Oslo, Norway; ^13^Institute of Clinical Medicine, University of Oslo, Oslo, Norway; ^14^Paediatric Unit, Department of Health Sciences, Meyer Children’s Hospital, University of Florence, Florence, Italy; ^15^Karoliniskiu Policlinic, Vilnius, Lithuania; ^16^Clinic of Children’s Diseases, Institute of Clinical Medicine, Medical Faculty of Vilnius University, Vilnius, Lithuania; ^17^Human Ecology Research Group, Department of Public Health, Institute of Health Sciences, Medical Faculty of Vilnius University, Vilnius, Lithuania; ^18^Paediatric Praxis Kurwerk, Burgdorf, Switzerland; ^19^Department of Paediatric Research, University of Oslo, Oslo, Norway

**Keywords:** choosing wisely, overtreatment, over-testing, survey, children

## Abstract

Studies and initiatives such as the “Choosing wisely” (CW) campaign emphasise evidence-based investigations and treatment to avoid overdiagnosis and overtreatment. The perception of the extent of medical overactivity among professionals and drivers behind are not well studied in the paediatric field.

**Aim:**

We aimed to investigate the physicians‘ opinion and clarify the main drivers regarding medical overactivity in member countries of the European Academy of Paediatrics (EAP).

**Methods:**

In this study, paediatricians, paediatric residents, primary care paediatricians, and family doctors treating children were surveyed in Norway, Lithuania, Ukraine, Italy, and Switzerland. Over-investigation was defined as “diagnostic work-up or referral that is unlikely to provide information which is relevant for a patient” and overtreatment was defined as “treatment that does not benefit or can harm more than benefit the patient.” The original questionnaire was developed in 2018 by a working group from the Norwegian Paediatric Association.

**Results:**

Overall, 1,416 medical doctors participated in the survey, ranging from 144 in Lithuania to 337 in Switzerland. 83% stated that they experienced over-investigation/overtreatment, and 81% perceived this as a problem. The majority (83%) perceived expectations from family and patients as the most important driver for overtreatment in their country. Other drivers for overuse were use of national guidelines/recommendations, worry for reactions, and reduction of uncertainty.

**Conclusion:**

This is the first study investigating knowledge and attitude toward medical overactivity in European countries. Despite different cultural and economic environments, the patterns and drivers of increased investigations and medicalisation are similar.

## Introduction

One of the most important commitments of a medical professional is to do no harm. This is a fundamental ethical concept starting from the first contact with a patient who aims for a thorough reasonable diagnostic evaluation, leading to relevant and evidence-based treatment options. Over-testing and overtreatment have become a growing concern worldwide. Published articles, books, and initiatives such as the “Choosing wisely” (CW) campaign, are bringing attention to this issue. Despite this, the awareness of the main causes of over-testing and medical overuse amongst clinicians remains limited.

Children are considered especially fragile, and the disease course can be fast-changing. This can reinforce a belief, that more is better, inducing excessive investigations or overtreatment (1). Currently, different paediatric international, national guidelines and recommendations are focussing on evidence-based health care. Initiatives such as “Choosing wisely” provide clinicians with a set of recommendations and references for the evidence-based diagnostics and treatment of various common conditions, such as bronchiolitis, dehydration, or head trauma (2). Various clinical decision tools, such as Paediatric Emergency Care Applied Research Network (PECARN), a paediatric head injury/trauma algorithm, Centor score (criteria for strep throat) or paediatric appendicitis risk calculator (pARC) could help physicians’ decision- making process and decrease the possibility of medical error, improving sensitivity and specificity in the diagnostic process (3–5).

There is limited knowledge regarding the drivers of over-investigation and overtreatment in paediatric care. Literature suggests that fear of litigation and to miss serious conditions may drive practices that minimise the space for mindful and patient-oriented evaluations, fear of negative criticism, and following outdated guidelines not reflecting current evidence-based knowledge. Time constrains may also drive medical overactivity, such as strict schedules and limited duration of visits at a general practitioner’s (GP) or primary paediatricians’ office. Others point toward expectations from families and patients, also from more senior colleagues. Financial reasons may also fuel overtreatment. Repeated office and emergency room (ER) visits might stimulate increased testing or unnecessary prescriptions. Despite these, knowledge of the most important drivers of overtreatment in paediatric medical service is insufficient.

The “Choosing wisely” working group of the European Academy of Paediatrics (EAP) aimed to investigate and clarify the physician’s opinions as well as the major drivers regarding overtreatment/over-investigation in Norway, Lithuania, Ukraine, Italy, and Switzerland—five European countries with distinct healthcare systems, historical and cultural backgrounds and economical statuses. We hypothesised, that contrasting cultural and economic backgrounds of involved countries as well as age and experience of a physician can be associated with the different attitude toward medical overuse.

## Materials and methods

In this study, we collected responses in a survey among paediatricians, paediatric residents, primary care paediatricians, and family doctors treating children. Overtreatment was defined as “treatment that does not benefit or can harm more than benefit the patient.” This term includes the prescription of all “unnecessary” drugs, including antibiotics, proton pump inhibitors, polypharmacy or other therapies. Over-investigation was defined as “diagnostic work-up or referral that is unlikely to provide information which is relevant for the patient”; this term includes either blood tests, specialists’ visits, unnecessary imaging and even hospitalisation. For this article, the two terms will be named with the inclusive term of “medical overuse.”

The original questionnaire addressing medical overuse was developed in 2018 by a working group from the Norwegian Paediatric Association (NPA) (BN, JS, KK, and KS) before the launch of the national CW campaign. In Norway, the original survey was sent out by email to all members of the NPA in June 2018. A reminder was then sent out three weeks later.

The survey was slightly shortened and translated for use in four other participating countries. Lithuania used a similar approach by email invitation during April–September 2020 as in Norway, but additionally invited participants through meetings in their society. From March to November 2021, Ukraine, Switzerland, and one region of Italy (Tuscany) distributed the survey to the members of their societies by email. In addition to emails, Switzerland also invited participants by newsletters sent out by the two national paediatric associations.

The initial questions in the survey for baseline characteristics were answered by all participants (Table 1). The participants were asked about their general opinion about medical overuse in their own country, at their working place and among their colleagues. They were asked to estimate how much of medical treatment and diagnostics in their own country they would regard as medical overuse selecting alternatives in an incremental scale from <10 to >50%. Lastly, they were asked about their own experience. Only those who confirmed medical overuse in their own practice were asked to grade potential drivers behind from specific alternatives on a Likert scale from 1 (do not agree) to 6 (completely agree).

**TABLE 1:** Participants in survey of overtreatment/over-investigation in five European countries.

	Norway	Lithuania	Ukraine	Italy	Switzerland	Total
	(n = 297)	(n = 144)	(n = 319)	(n = 319)	(n = 337)	(n = 1,416)
Female, n (%)*						
	166 (56)	124 (86)	291 (91)	223 (72)	197 (62)	1001 (71)
**Age group, n (%)^†^**						
<35 years	56 (19)	73 (51)	81 (25)	74 (23)	43 (13)	327 (23)
35–50 years	118 (40)	38 (26)	119 (37)	117 (37)	137 (42)	529 (38)
>50 years	119 (41)	33 (23)	119 (37)	126 (40)	144 (44)	541 (39)
**Place of work, n (%)^‡^**						
Hospital	229 (77)	93 (65)	72 (23)	221 (69)	80 (24)	695 (49)
Clinical outside hospital	22 (7)	37 (26)	220 (69)	89 (28)	224 (66)	592 (42)
Academic/admin./other	45 (15)	14 (10)	27 (8)	9 (3)	33 (10)	128 (9)
**Percentage clinical work, n (%)^§^**						
<50	56 (19)	25 (17)	53 (17)	11 (3)	43 (13)	188 (13)
50–99	53 (18)	83 (58)	171 (54)	192 (60)	211 (65)	710 (51)
100	183 (63)	36 (25)	95 (30)	116 (36)	73 (22)	503 (36)

*Missing gender: n = 33.

^†^Missing age group: n = 19.

^‡^Missing place of work: n = 1.

^§^ Missing percentage of clinical work: n = 15.

Written informed consent for participation was not required and all participant answers were anonymous, so ethical permission was not required.

## Statistics

The data were stored in Microsoft Excel and analysed using Stata (StataCorp. 2019. *Stata Statistical Software: Release 16*. College Station, TX: StataCorp LLC.). Categorical variables were expressed as percentages. We dichotomised the answers to the questions of “overdiagnosis in your country” and “overdiagnose yourself” in order to study participants’ characteristics associated with answering “yes” vs “unsure/no.” Finally, predictors for responding “yes” were analysed by logistic regression. Predictors with a *p*-value of <0.10 were used in a final multivariate logistic regression.

## Results

In total, 1,416 participated in the survey ranging from 144 in Lithuania to 337 in Switzerland (Table 1). Females dominated (71%), 39% were >50 years and 38% between 35 and 50 years. Forty-nine percent worked in hospital settings, ranging from less than 25% in Ukraine and Switzerland to 77% in Norway. Only 13% worked less than half of their time in patient care (Table 1).

**FIGURE 1:**
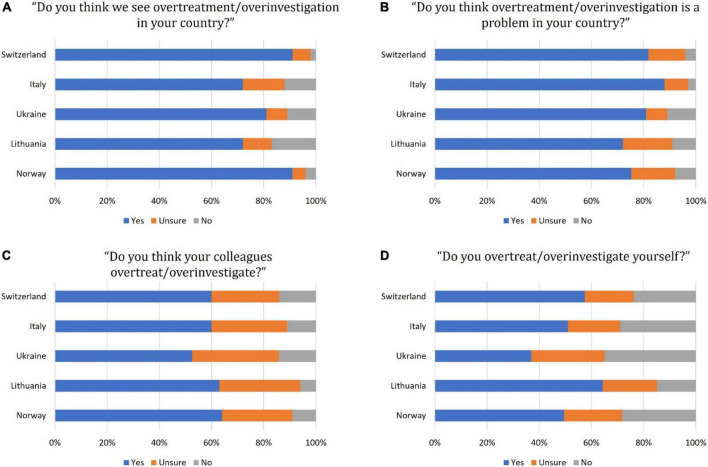
Responses among different countries to the “Choosing wisely” survey questions **(A–D)**. A stacked bar chart represents responses in percentage on an incremental scale from 0 to 100% from one country all respondents.

Overall, 83% of respondents in all countries perceived overtreatment in their country, ranging from 72% in Lithuania and Italy to 81% in Ukraine, and 91–92% in Norway and Switzerland (Figure 1A). The remainders responded “unsure” or “no” in similar percentages. A slightly lower percentage (81%) responded “yes” to the question “Is it a problem” (Figure 1B displays individual countries). Fifty-eight percent stated that their colleagues overtreated (Figure 1C).

**FIGURE 2:**
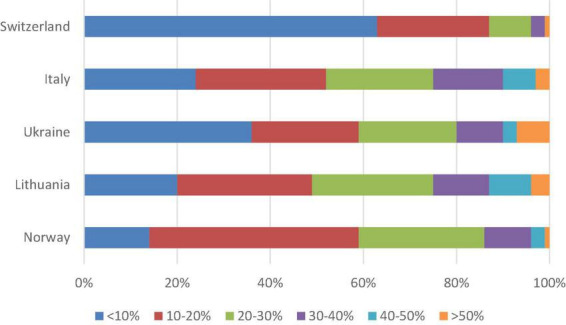
Responses among different countries to the question “How much of medical practice in your country by your opinion is overtreatment/overinvestigation?”. A stacked bar chart represents responses in percentage on an incremental scale from 0 to 100% from one country all respondents.

When asked about their individual practice, 51% stated that they overtreatedsame. In Ukraine, 37% responded “yes” to this question, whereas 65% of Lithuanian doctors admitted doing so (Figure 1D for country-wise differences). The extent of medical overactivity was estimated to be <10% by 35% of the participants, 10–20% by 29%, and >20% by 37% of the responders. We found a substantial variation across the countries in these estimates, and particularly participants from Italy and Lithuania tended to estimate the extent higher than the three other countries (Figure 2).

**FIGURE 3:**
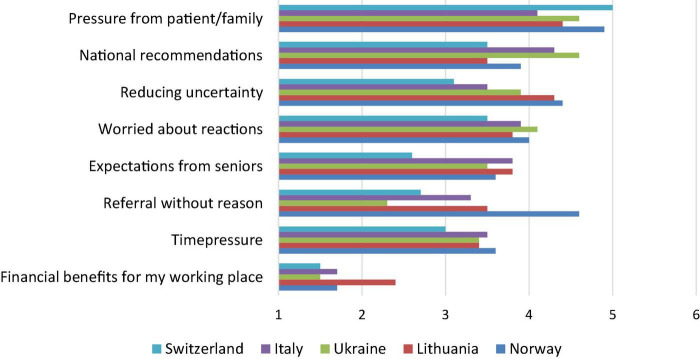
Responses among different countries to the question “What are by your opinion the main reasons that you overtreat/overinvestigate (n = 909-921)?” Agree-disagree scale data, where 1 = totally disagree, 2 = partially disagree, 3 = slightly disagree, 4 = slightly agree, 5 = partially agree, and 6 = totally agree.

The highest ranked driver behind medical overuse was expectations from family and patients, to which 83% of the doctors agreed and with minimal variation across the countries (Figure 3). National guidelines/recommendations, worry for parents’ attitudes/reactions, and reduction of uncertainty were also ranked as major drivers. Of lesser importance were time pressure, expectations from seniors, and referral practices. Financial reasons were ranged the least important.

The main predictor of perceiving medical overuse in their country was country-dependent, i.e., Norway and Switzerland had significantly higher odds than the other three countries. Doctors younger than 50 were more concerned about medical overactivity than colleagues >50 years (adjusted odds ratio 0.58, 95% CI 0.43–0.79) (Table 2), while the opinions were similar in males and females. The predictors for “overtreating yourself” were very similar as for the first question of “medical overuse in your country” (data not shown).

**TABLE 2:** Predictors for perceiving overtreatment/over-investigation (n = 1376 participants).

	Overtreatment/-investigation		Unadjusted odds ratio (95% CI)	Adjusted odds ratio (95% CI)*	*P*-value
	Yes (n = 1, 169)	No/unsure (n = 244)			
**Country, n (%)**					
Norway	268 (91)	27 (9)	Ref.	Ref.	
Lithuania	104 (72)	40 (28)	0.26 (0.15–0.45)	0.22 (0.13-0.40)	<0.001
Ukraine	258 (81)	60 (19)	0.43 (0.27–0.70)	0.41 (0.25–0.69)	0.001
Italy	230 (72)	89 (28)	0.26 (0.16–0.41)	0.25 (0.15–0.40)	<0.001
Switzerland	309 (92)	28 (8)	1.11 (0.64–1.93)	1.17 (0.65–2.11)	0.59
**Sex, n (%)**					
Female	818 (82)	181 (18)	Ref.	Ref.	
Male	327 (86)	54 (14)	1.34 (0.96–1.86)	1.15 (0.80–1.66)	0.46
**Age group, n (%)**					
<35 years	274 (84)	53 (16)	Ref.	Ref.	
35–50 years	454 (86)	74 (14)	1.19 (0.80–1.74)	0.93 (0.62–1.39)	0.73
>50 years	426 (79)	113 (21)	0.72 (0.51–1.05)	0.55 (0.38–0.82)	0.003
**Main workplace, n (%)**					
Hospital	565 (82)	128 (18)	Ref.	NA	
Clinical outside hospital	500 (85)	91 (15)	1.24 (0.93–1.67)		
Academic/admin./other	104 (81)	24 (19)	0.98 (0.61–1.59)		
**Clinical position, n (%)**					
<50%	156 (83)	32 (17)	Ref.	NA	
50–99%	597 (84)	113 (16)	1.08 (0.70–1.67)		
100%	406 (81)	94 (19)	0.89 (0.57–1.38)		

*Adjusted for country, sex and age group.

## Discussion

This is the first study showing opinion and personal insights on medical overuse of medical professionals working in the field of paediatrics from European countries. Despite the difference in countries’ cultural and economic background, most respondents noted overtreatment in their country and defined it as a problem. After analysis, we observed that all countries specified family and/or patients expectations as the main driver causing over-investigation or overtreatment with financial reasons being the least important.

Medical investigations provide crucial information leading to a decision on proper treatment options. However, over-testing may potentially mislead in the diagnostic process with false positive results leading to a misdiagnosis meaning that a person has a “disease” for a current condition that would cause him/her no harm if left undetected or without specific treatment (6–8). As an example, some minimal changes in routine diagnostic tests (e.g., complete blood count) in an asymptomatic individual can lead to anxiety and an overflow of unnecessary additional tests (6, 7). With the increasing accessibility of testing, which is more rapid and in some cases cheaper than before, and, importantly, more sensitive for milder conditions, the field of paediatrics is especially vulnerable to widespread testing leading to diagnoses that might disserve the patient. The rapid dynamics of different paediatric diseases, worrisome symptoms despite well general appearance and reassuring physical examination, parents’ concerns, and forms of cognitive bias of a treating physician, systemic and public pressure are boosting the cascade of unnecessary diagnostic and treatment events (9). Additionally, in the time of COVID-19, overtreatment due to fear of COVID-19 itself may have led to a boost of remote consultations and lack of proper diagnostics (10).

The scientific and clinical data suggest that medical overuse can be associated with different cultural backgrounds, different medical systems, local protocols, or implemented guidelines and economic development (11). Our study invited medical professionals in five European countries with clear variation in medical systems and funding, e.g., the highest spending on healthcare as a share of GDP being Switzerland and Norway (11.3 and 10.5%, respectively), followed by Italy (8.7%), Ukraine and Lithuania (both 7%) (12, 13). Despite this, no dramatic differences were found between the five countries and the majority of respondents in all these countries defined overtreatment and over-investigation as a problem in their country. However, more physicians in Switzerland and Norway (having the highest health spending per capita) perceived medical overuse compared to the three countries with lower health spendings (12, 13).

Interestingly, a higher percentage of Italian, Lithuanian, and Ukrainian physicians expressed their uncertainty toward overtreatment/over-testing. Those variations could be explained due to differences in the development of the medical system as well as different protocols and attitudes toward specific guidelines, and compliance to them. Moreover, the implementation of programs such as “Choosing wisely” or antibiotic stewardship programs could have been increasing the knowledge and awareness in some countries. The “Choosing wisely” campaign launched in 2012 in the United States, was partially known in Norway, Switzerland, and Italy at the time of survey, with only initial steps in Ukraine and Lithuania (14).

Primary care has often been attributed the biggest role in medical overuse. Considering limited time for each patient, the risk of frequent revisits, and somewhat moderate accessibility to specific investigation methods, several studies show primary care physicians’ role in an increased prescription for diseases such as viral respiratory infections or asthma (15–17). Interestingly, half of our respondents were physicians at university hospitals and 82% of them did perceive overtreatment in their settings (data not shown). Children who are referred to the hospitals’ paediatric ER may be more likely to get additional testing, such as CT scans in case of head trauma (18) and blood tests for viral diseases (1). Nevertheless, it is important to emphasise that ER settings and staff can influence over-testing and overtreatment, e.g., less testing and less imaging is associated with exclusively paediatric centres opposing non-paediatric ones (19), meanwhile, data show that paediatric emergency physicians may perform fewer diagnostics tests compared to general paediatricians working in ED (20). Furthermore, repeated visits to ER can lead to a higher hospitalisation rate (21). After such admission, paediatric patients do risk getting multiple repeated testing, interventions that do not contribute significantly to the diagnosis, as well as change in treatment strategies with an increased length of stay (22–24). The shift toward more outpatient care and shorter hospital stays may also lead to more reliance on additional testing.

Despite that, all five countries have different historical and cultural characteristics, the majority of perceived drivers had striking similarities across the countries. Parental concern, anxiety, and pressure could stimulate over-testing with further excessive treatment (25). Not surprisingly, in our study, we observed that parental concern was the most important driver of overtreatment in all the countries. However, different publications show that parental expectations are not always well understood due to lack of time or miscommunication (26, 27). Moreover, with regard to repeated visits or questions, a physician may assume that parents ask for additional testing or treatment which is not always the case (26). The significant pressure from a society and public media emphasising children as vulnerable boosts parental distress and pushes a physician toward a higher testing rate and medicalisation due to fear to miss a specific diagnosis as well as concerns about public opinion and reaction. In this study, we confirmed that worry for reactions/attitudes was ranked high as a driver by all physicians across the countries. The fear of error or misdiagnosis as well as fear of public reactions could be reduced with implementing and following local or national recommendations and guidelines. However, the majority of the physicians responded that national recommendations could contribute to overtreatment/over-investigation. It must be noted that there could be interpretations and different attitudes toward guidelines, protocols or policies which could significantly influence testing or prescription behaviour. Some studies show that adherence to the protocols could contribute to increased testing (28). In contrast, non-compliance or not knowing the newest guidelines could influence medical overuse (28, 29). Education and frequent updates on recommendations could diminish over-testing and improve awareness on medical overuse.

Various data show that physicians’ cognitive bias and fear of a medical error are one of the influential and frequently encountered factors causing medical overuse (30). The intolerance of uncertainty can be associated with excessive testing (31). Physicians can be worried about not having an answer to patients prolonged symptoms or new complaints. This can result in an over-investigation and increased referral to more specialised care. Most of our respondents emphasised that uncertainty drives over-testing and overmedication. Moreover, we noted that a higher percentage of younger respondents experienced overtreatment/over-testing which could be a “compensation mechanism” due to lack of experience or knowledge. Increasing the number of investigations increases the possibility of misdiagnosis, thus, unnecessary treatment with a potential harm to a patient and even society (32, 33).

Certainly, our study has some limitations. First, more countries can be included into the study to contribute to a better understanding the differences between countries leading to better decisions in policy making and deimplementation strategies (34). Another issue is that none of the included countries are unaware of the “Choosing wisely” campaign. Thus, more countries with different experiences on this topic could be surveyed to have a broader picture of paediatric overtreatment and over-testing. Another limitation is that this study was conducted via online questionnaires and that low response rates could create a selection bias among respondents. In addition, the survey was distributed, and data were collected at different timepoints, i.e., some of the countries collected data before the COVID-19 pandemics (e.g., Norway), meanwhile others filled the questionnaire during the pandemics, thus, it could have influenced on some responses. Moreover, in the additional comment section some of the respondents indicated other reasons for medical overuse. Those reasons were not included in the primary survey. So, some specific country-dependant reasons due to different cultural or economical background exist and should be addressed in the future. In this questionnaire, the results obtained regarding the medical overuse in its inclusive and general value, without giving more details about different kind of over-diagnostic and over-treatment.

Nevertheless, the biggest strength of our study is that this is the first study on knowledge and attitude regarding medical overuse including different physicians working with children in five European countries. There has been limited data on paediatricians’ opinions related to over-testing and overtreatment, especially among those working in hospitals. Thus, our study contributes to the worldwide data concerning medical overuse. Another strength is our findings showing important drivers, such as expectations from family and patients, worry for their attitudes/reactions, and reduction of uncertainty, which correspond to the data from other study groups. It reinforces the necessity of a wider spread of awareness of medical overuse and introduction and validation of different recommendations internationally and nationally.

In conclusion, our study is the first study including opinion of the physicians’ experience on over-testing and overtreatment in European countries. We demonstrated that despite cultural and economic differences the patterns and drivers of increased investigations and medicalisation are similar across the included countries.

## Data availability statement

The raw data supporting the conclusions of this article will be made available by the authors, without undue reservation.

## Ethics statement

Ethical review and approval was not required for the study on human participants in accordance with the local legislation and institutional requirements. Written informed consent for participation was not required for this study in accordance with the national legislation and the institutional requirements.

## Author contributions

KS, KK, BN, and JS: original questionnaire creation, survey distribution, and data collection (Norwegian part). YG, MM, and IR: survey translation, adjustment, distribution, and data collection (Ukrainian part). SS and CW: survey translation, adjustment, distribution, and data collection (Swiss part). ST: survey translation, distribution, and data collection (Italian part). LJ, SD, and AV: survey translation, adjustment, distribution, and data collection (Lithuanian part). KS: study supervision, data summary, and statistical analysis. LJ and KS: manuscript drafting. All authors contributed to manuscript editing, read and agreed with the final version.
