# Supplementary material for: Overuse of medical care in paediatrics: A survey from five countries in the European Academy of Pediatrics
Source: Front Pediatr. 2022 Sep 13;10:945540. doi: 10.3389/fped.2022.945540 (PMC9513058; doi:10.3389/fped.2022.945540)
Supplement: Supplementary file 1 [file Data_Sheet_1.PDF]

QUESTBACK - “CHOOSING WISELY”

INTRODUCTION:

Everything appears on page 1

Introduction to the study: The Paediatric Association and the Norwegian Medical Association are now working on a campaign against over-treatment and over-investigation. The campaign is modelled by Choosing Wisely, and in Norwegian called "Make Wise Choices".

Before fully starting the campaign, we ask you to take part in a survey.

The purpose is to obtain knowledge about doctors' attitudes towards over-treatment and over-examination, and about under-treatment and under-examination. The survey will form the basis for evaluating the effect of the campaign "Make Wise Choices".

Estimated response time is 6-7 minutes.

To respond to the survey, use this link:

https://response.questback.com/Legeforeningen/eczvewivns

With best regards

Ketil Størdal

Head, Norwegian Paediatric Association.

Gender

Woman, man

How old are you?

<35, 35-50, > 50

Where is your main workplace?

Specialist health service in hospitals, general health service, private practice specialist, administration, academic position, research, pensioner, other (*free-text field after «other»*)

How much of your working time do you have in a clinical position?

<50%, 50-99%, 100%

MAIN SECTION:

Definitions and Questions 1 on page 2.

Definition of over-treatment: Treatment that does not benefit or may cause more harm than benefit.

Definition of over-investigation: Diagnosis or referral that reasonably certain will not provide important information.

Question 1: Do you think we see over-treatment / over-reporting in the Norwegian health care system?

Yes, no, unsure

If yes or uncertain answer: continue with question 2. If answer no: continue with question 8

Questions 2-4 on page 3.

Question 2: Do you experience over-treatment / over-reporting in the Norwegian health system as a problem?

Yes, no, unsure

Question 3: Do you think it is over-processed / over-investigated at your department / workplace?

Yes, no, unsure

Question 4: Do you find yourself over-treating / over-investigating?

Yes, no, unsure

If yes or uncertain answer: continue with question 5. If answer no: continue with question 6.

Question 5 on page 4.

Question 5: What are the reasons you are over-treating / over-investigating? (*Rate the statements below from: totally disagree, partially disagree, slightly disagree, slightly agree, partially agree, totally agree*)

Great time pressure

The treatment / procedure is expected by (superior) colleague

Referred to my practice on a weak basis

Pressure from patient / relatives

Anxious for retaliation / complaint

It reduces uncertainty

Always follow national guidelines / supervisor

Financially beneficial in my workplace

Questions 6-7 on page 5.

Question 6: What proportion of assessment / treatment at your department / practice would you estimate is over-examination / over-treatment?

<10%, 10-19%, 20-29%, 30-39%, 40-49%,> 50%

Question 7: Do you think your colleagues are over-treating / over-investigating?

Yes, no, unsure

Definitions and Questions 8 on page 6.

Definition of under-treatment: When the patient has a condition where the indications are clear, and the treatment has documented efficacy and where the patient does not receive the necessary health care.

Definition of Sub-Inquiry: When a patient is not given a diagnosis or referral that could reasonably provide meaningful information.

Question 8: Do you think we see under-treatment / under-examination in the Norwegian health care system?

Yes, no, unsure

If yes or uncertain answer: continue with question 9. If answer no: continue with question 13.

Questions 9-12 on page 7.

Question 9: Do you experience under-treatment / under-examination in the Norwegian health system as a problem?

Yes, no, unsure

Question 10: Do you think it is under-processed / under-investigated at your department / workplace?

Yes, no, unsure

Question 11: Who / what groups do you think are under-processed / under-researched? (*Free text field*)

Question 12: What do you think are the reasons for under-processing / under-investigation? (*Rate the statements below from: totally disagree, partially disagree, slightly disagree, slightly agree, partially agree, totally agree*)

Great time pressure

Lack of compliance with patient / relatives

Resistance of the patient / next of kin

Always follow national guidelines / supervisor

It reduces your spending / cost is too great

The offer is not available at my place of work

Question 13 on page 8.

Question 13: Do you know / have heard of the campaign: "Making wise choices" / "Choosing wisely"?

Yes No

If answers yes: continue to question 14. If answers no: continue to Feedback.

Questions 14-19 on page 9.

Question 14: Where did you hear about the campaign? (*More answers possible*)

International trade journals, NBF meetings, National journals including Paidos,

Today's medicine, social media, via colleagues, other (*free-text field after "other"*)

Question 15: Consider the following statements: (*Rate the statements below: Strongly Disagree, Partially Disagree, Slightly Disagree, Slightly Agree, Partially Agree, Strongly Agree.*)

The campaign "Make wise choices" will be based on evidence-based material.

The "Make wise choices" campaign will reduce costs.

The "Make wise choices" campaign will promote good communication and information between doctor and patient.

The "Make wise choices" campaign will reduce under-processing / under-investigation.

The "Make wise choices" campaign will reduce over-processing / over-reporting.

Question 16 Do you think this campaign will change your practice?

Yes, no, uncertain

Question 17: As part of the campaign "Make wise choices", the Norwegian Paediatric Association has prepared 5 "wise choices", do you know these?

Yes, no, unsure

Question 18: Do you think the 5 "wise choices" from the Paediatric Association will have an impact on your practice?

Yes, no, unsure

Question 19: Do you think the 5 "wise choices" from the Paediatric Association will have an impact on the practice of others?

Yes, no, unsure

FEEDBACK:

Feedback on page 10.

We would like feedback on the five "wise choices" that have been prepared. If you have any suggestions for new recommendations, please also comment on this: (*free-text field*)

Short conclusion by choice: Thank you for participating.
